# Adjunctive Use of VGH4 for Moderate-to-Severe Atopic Dermatitis: A Randomized, Double-Blind, Placebo-Controlled Crossover Pilot Trial

**DOI:** 10.3390/life16040680

**Published:** 2026-04-16

**Authors:** Ying-Ju Liao, Ta-Peng Wu, Chou-Cheng Lai, Yen-Ying Kung, Cheng-Hung Tsai, Yun-Ting Chang, Chih-Chiang Chen, Ching-Mao Chang, Shinn-Jang Hwang, Fang-Pey Chen

**Affiliations:** 1 Institute of Traditional Medicine, College of Medicine, National Yang Ming Chiao Tung University, Taipei 112, Taiwan; 2 Center for Traditional Medicine, Taipei Veterans General Hospital, Taipei 112, Taiwan; 3 School of Medicine, National Yang-Ming University, Taipei 112, Taiwan; 4 Division of Infectious Diseases, Department of Pediatrics, Taipei Veterans General Hospital, Taipei 112, Taiwan; 5 School of Chinese Medicine, College of Medicine, National Yang Ming Chiao Tung University, Taipei 112, Taiwan; 6 Department of Dermatology, Taipei Veterans General Hospital, Taipei 112, Taiwan; 7 Faculty of Medicine, School of Medicine, National Yang Ming Chiao Tung University, Taipei 112, Taiwan; 8 Department of Dermatology, National Yang Ming Chiao Tung University, Taipei 112, Taiwan; 9 Institute of Clinical Medicine, National Yang Ming Chiao Tung University, Taipei 112, Taiwan; 10 Family Medicine Division, Chang Bin Show Chuan Memorial Hospital, Changhua County 505, Taiwan

**Keywords:** atopic dermatitis, traditional Chinese medicine, Chinese herbal medicine, randomized controlled trial, quality of life

## Abstract

Moderate-to-severe atopic dermatitis (AD) requires safe, long-term management strategies to complement conventional pharmacotherapy. This study evaluated the efficacy and safety of VGH4, a standardized multi-herb traditional Chinese medicine (TCM) formula, as an adjunct to standard care. In a randomized, double-blind, placebo-controlled crossover pilot trial, 19 patients with moderate-to-severe AD (SCOring Atopic Dermatitis Index (SCORAD) ≥ 25) received VGH4 or placebo for 6 weeks, separated by a 2-week washout. Primary outcomes assessed disease severity (SCORAD), while secondary outcomes included quality of life (DLQI/CDLQI) and safety. Eighteen patients completed the study. VGH4 yielded a median within-patient SCORAD reduction 10.2 points greater than placebo (*p* = 0.054). The primary endpoint did not reach statistical significance at the α = 0.05 level (*p* = 0.054); nevertheless, the observed magnitude of improvement exceeded the established minimal clinically important differences (MCIDs). The subjective SCORAD component showed a significant between-treatment difference favoring VGH4 (*p* = 0.015), and a statistically significant improvement in quality of life was also observed in adult patients (*p* = 0.023). In conclusion, VGH4 was generally well tolerated in this short-term pilot trial, with no serious adverse events, and showed preliminary signals of possible benefits in patient-reported outcomes as an adjunct therapy. These exploratory findings warrant confirmation in larger, adequately powered trials.

## 1. Introduction

Atopic dermatitis (AD) is a common chronic skin disorder characterized by inflammation that often begins in early childhood and affects up to 20% of children worldwide. Its prevalence in Taiwan is around 6–10%, signifying a considerable healthcare burden [1,2]. AD leads to intense pruritus, eczematous lesions, xerosis, and skin barrier dysfunction, collectively leading to an impairment in quality of life (QoL) [3]. Regarding the accessibility of standard therapies, challenges remain in the long-term management of AD. First-line topical anti-inflammatory treatments, including topical corticosteroids and topical calcineurin inhibitors, may be associated with treatment-related concerns during prolonged or repeated use, such as cutaneous atrophy and local stinging or burning sensations; diminished responsiveness with continued topical therapy has also been described [4]. Biologics and Janus kinase (JAK) inhibitors have expanded the systemic treatment options for AD; however, their use is still constrained by cost and accessibility, and oral JAK inhibitors require individualized risk assessment and ongoing monitoring [5,6,7]. In this context, some patients with chronic inflammatory skin conditions consider traditional Chinese medicine (TCM) as a complementary approach. TCM has a long history of use for the treatment of eczema, and controlled studies have shown that it may help adults and children with AD-related outcomes by reducing the need for steroids; however, the overall evidence remains limited [8,9,10].

In TCM theory, AD is conceptualized as a manifestation of systemic imbalance and is treated using a whole-person approach. Commonly implicated TCM patterns include “spleen deficiency with dampness” and “wind–heat”, and treatment may involve multi-herb formulas, acupuncture, and dietary therapy [11]. Meta-analyses indicate that certain TCM remedies may mitigate eczema severity and enhance QoL while maintaining acceptable safety profiles [8,9]. Furthermore, stringent hospital-led randomized controlled trials (RCTs) have demonstrated the benefits of oral TCM in several chronic illnesses, including globus sensation and chronic hepatitis C, using double-blind, placebo-controlled designs [12,13,14,15]. We developed the VGH4 herbal mixture for patients with moderate-to-severe AD. The formulation was informed by empirical prescribing patterns derived from Taiwan’s National Health Insurance Research Database (NHIRD) and established TCM formulations for AD. Xiao-Feng-San (XFS) and Dang-Gui-Yin-Zi (DGYZ) are commonly used traditional Chinese medicine (TCM) formulas for eczema, including atopic dermatitis, and constitute the principal components of VGH4 [16]. In the TCM framework, XFS is frequently prescribed for acute or subacute presentations characterized by “wind” and “damp-heat,” whereas DGYZ is used for chronic presentations attributed to “blood deficiency” and “wind dryness.” To further address pruritus and damp-heat-related features, VGH4 also includes two single herbs widely used in TCM dermatology, Bai-Xian-Pi (*Dictamnus dasycarpus Turcz.*) and Di-Fu-Zi (*Kochia scoparia* (L.) Schrad.), which are commonly added to eczema prescriptions to clear damp-heat and relieve itching [17]. Prescription network analyses have identified XFS, in combination with Bai-Xian-Pi and Di-Fu-Zi, as a core treatment cluster for eczema, and have suggested that these adjunct herbs may augment the anti-inflammatory and anti-allergic activities attributed to XFS [18]. VGH4 was formulated by pairing XFS (targeting acute “wind–damp–heat” patterns) with DGYZ (targeting chronic “blood deficiency” patterns) and these adjunct herbs, with the aim of addressing both acute and chronic clinical manifestations of AD. The dosage of each component was selected to reflect clinical use while minimizing overlap, and the composition of each dose is summarized in Table 1.

Here, we report a pilot, randomized, double-blind, placebo-controlled, crossover trial evaluating the efficacy, safety, and tolerability of VGH4 in patients with moderate-to-severe AD.

## 2. Materials and Methods

### 2.1. Patient Enrollment

This study was conducted at the Departments of Dermatology, Pediatrics, and Traditional Chinese Medicine at Taipei Veterans General Hospital (Taiwan) from January 2021 to December 2022. Eligible patients were consecutively recruited and met all of the following inclusion criteria: (1) age 6–30 years, with a clinical diagnosis of atopic dermatitis according to the Hanifin and Rajka criteria [19]; (2) SCOring Atopic Dermatitis Index (SCORAD) ≥ 25, indicating moderate-to-severe disease [20]; and (3) inadequate response to standard first-line treatments (such as topical steroids and moisturizers). Patients were excluded if they met any of the following criteria: (1) presence of an active skin infection (e.g., bacterial impetiginization); (2) use of systemic or topical therapies that could affect AD severity within the past 4 weeks (including systemic corticosteroids, immunosuppressants, biologics, phototherapy, systemic antibiotics, Chinese herbal medicines, or acupuncture); and (3) pregnancy or breastfeeding.

### 2.2. Ethics Approval

The study protocol was approved by the Institutional Review Board of Taipei Veterans General Hospital, Taiwan (IRB No. 2019-02-017A). Written informed consent was obtained from all participants. For participants who were minors at the time of enrollment, written informed consent was obtained from their parents or legal guardians, and assent was also obtained from the participants. The study was registered at ClinicalTrials.gov (NCT04686955).

### 2.3. Study Intervention

**Figure 1:**
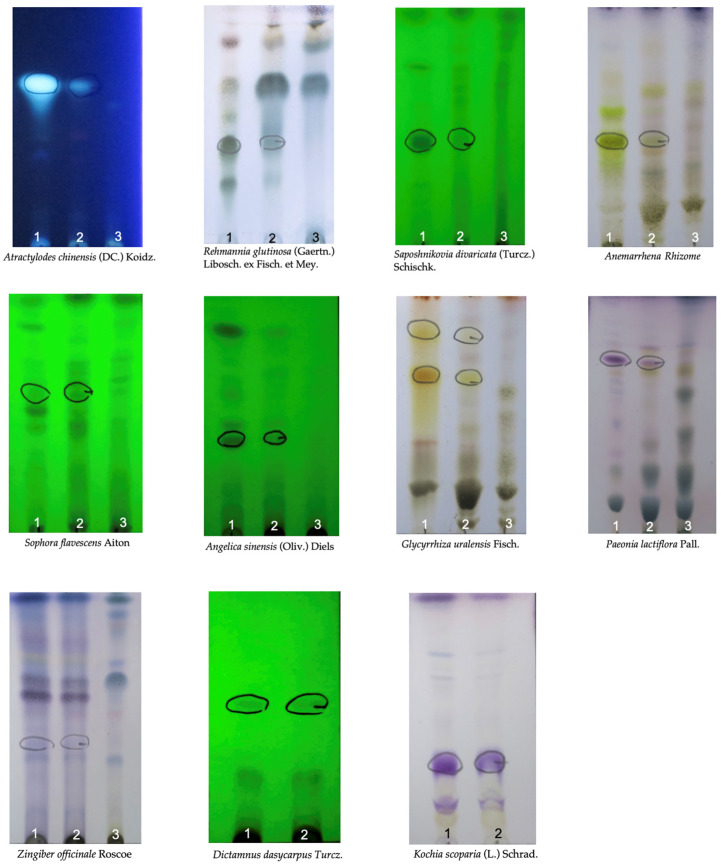
Thin-layer chromatography (TLC) fingerprint analysis used for the chemical characterization and identity testing of VGH4 products. Samples analyzed per well: [1] controlled medicinal herbs, [2] test samples, and [3] negative controls. The colored markings and circled areas indicate the corresponding reference compounds and characteristic bands for identification.

The experimental medication, VGH4, was administered as granules at a dose of 4.1 g per sachet. Table 1 shows that each dose included about 2.0 g of Xiao-Feng-San, 1.2 g of Dang-Gui-Yin-Zi, 0.4 g of Bai-Xian-Pi, and 0.5 g of Di-Fu-Zi. The placebo granules were designed to appear, taste, and smell similar to VGH4. They were formulated with inert starch-based fillers, colored with caramel, and supplemented with a small amount (1%) of VGH4 granules to give them an herbal taste. Both VGH4 and the placebo were produced by Ko Da Pharmaceutical Co. in Taoyuan, Taiwan, in accordance with Good Manufacturing Practices (GMPs). Thin-layer chromatography (TLC) fingerprint tests were performed as part of routine quality control (QC), according to the manufacturer’s procedure (Figure 1). Manufacturer QC specifications also include microbiological limits, heavy metals (As, Pb, Cd, Hg), and accelerated stability testing for the finished VGH4 product and matching placebo (Supplementary Files S1 and S2). Each medication was packaged in an identical foil packet. Patients were told to mix each dose with about 100 mL of warm water and consume it immediately, with dosing adjusted by age. For example, children aged 6 to 11 received 1 sachet per day, adolescents aged 12 to 17 received two sachets per day, and adults aged 18 and older received three sachets per day.

### 2.4. Study Design

**Figure 2:**
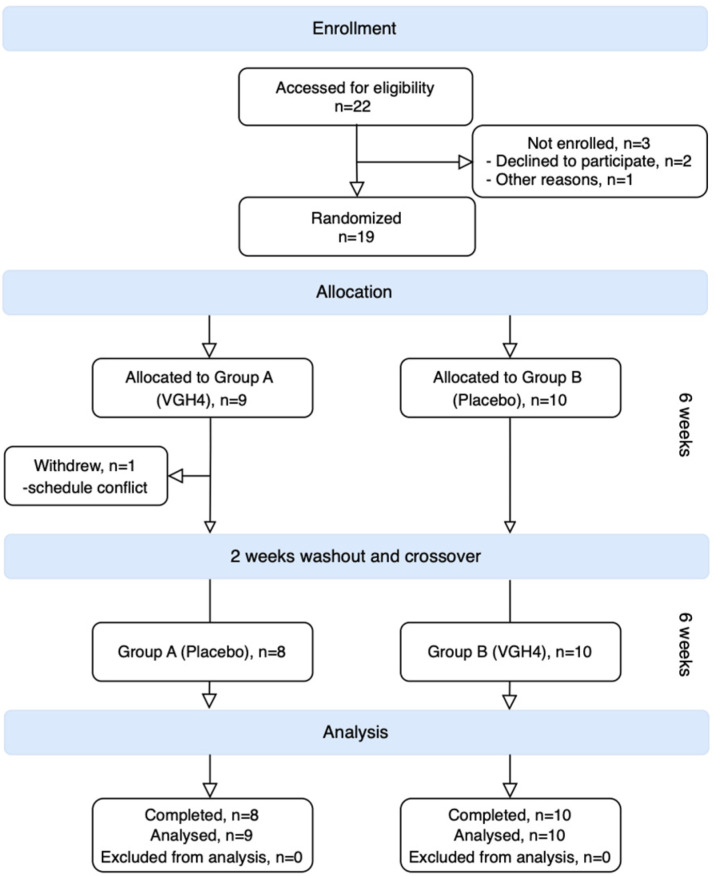
CONSORT flow diagram for the randomized controlled crossover pilot trial of VGH4 versus placebo in patients with atopic dermatitis.

This was a randomized, double-blind, placebo-controlled crossover pilot trial. Enrolled patients were randomly assigned, in a 1:1 ratio, to one of two groups: Group A received VGH4 in the first 6-week period, followed by placebo in the second 6-week period, whereas Group B received placebo first, and then VGH4 in the second period. A 2-week washout period (with no study medication) separated the two treatment periods to minimize carryover effects (Figure 2). Randomization was performed using a computer-generated permuted block sequence prepared by an independent statistician. Allocation was concealed via sequentially numbered, opaque, sealed envelopes that were opened by the dispensing pharmacist only after participant enrollment. Patients, investigators, and outcome assessors remained blinded to the treatment sequence throughout the study.

Clinic visits were scheduled every 3 weeks during each treatment period for assessments. All participants continued standard baseline therapy for AD during the trial: they were instructed to apply emollients 3–4 times daily, to use a low-potency topical steroid on active lesions twice daily [21], and to take oral antihistamines once daily. The same products used at baseline were maintained throughout the trial.

### 2.5. Outcome Measurements

The SCORAD (SCOring Atopic Dermatitis Index) was the primary outcome for quantifying eczema severity [20]. SCORAD evaluations were performed at baseline and throughout each scheduled follow-up visit (every 3 weeks) to assess changes during each therapy period. The secondary outcome was QoL, which was assessed via the Dermatology Life Quality Index (DLQI) for patients aged 16 years and over and the Children’s DLQI (CDLQI) for patients under 16 years, at baseline and at the end of the study. Systemic type 2 inflammatory biomarkers (total IgE, absolute eosinophil count, and eosinophil cationic protein (ECP)) and safety laboratory parameters (blood urea nitrogen (BUN), creatinine, alanine aminotransferase (ALT), and aspartate aminotransferase (AST)) were measured at baseline, week 6, and week 14.

### 2.6. Statistical Analysis

A minimum sample size of 17 patients completing both treatment periods was estimated to yield 80% power (two-tailed α = 0.05) to detect a within-patient difference in SCORAD improvement, predicated on an anticipated median SCORAD change of approximately −18 points in paired observations; baseline values were generated from a normal distribution with a mean of 50 and a standard deviation of roughly 15 (calculated through Monte Carlo simulation with 10,000 iterations). We analyzed the data on an intention-to-treat basis. Continuous variables were expressed as median [interquartile range], whereas categorical variables were expressed as number (%). We used the Wilcoxon rank-sum test for continuous variables and Fisher’s exact test for categorical variables to compare baseline characteristics between the two randomized sequence groups. The primary efficacy analysis used the Wilcoxon signed-rank test to assess the change in SCORAD between VGH4 and placebo in the same participants. Similar paired comparisons (or non-parametric equivalents) were utilized for secondary outcomes (DLQI and CDLQI) and laboratory measures. All statistical tests were two-tailed, and *p*-values less than 0.05 were deemed statistically significant.

## 3. Results

### 3.1. Participant and Baseline Characteristics

Nineteen patients were enrolled in the study between 2021 and 2022. The median age was 18.6 years (range 6–28 years), and 42% (8/19) were male. Eight participants were under 16 years of age. All patients had longstanding, relapsing moderate-to-severe AD (baseline median SCORAD score was 53, interquartile range [IQR] ~45–60), and all participants were of Asian ethnicity. Before enrollment, nearly all had been managing their disease with intermittent topical corticosteroids; none were on systemic immunosuppressants or biologics at baseline (often due to contraindications or a personal preference to avoid systemic therapies). The baseline demographic and clinical characteristics were comparable between the two randomization groups (Group A and Group B), with no significant differences in age, sex, or baseline disease severity (*p* > 0.3 for all; see Table 2). One patient withdrew from the study after completing the first treatment period (due to scheduling conflicts), leaving a total of eighteen patients (95%) who completed both crossover periods and were included in the efficacy analysis (Figure 1). Patients reported taking approximately 95% of the dispensed doses, as confirmed by sachet-count audits.

### 3.2. Primary Outcome: SCORAD (SCOring Atopic Dermatitis Index)

VGH4 therapy was associated with a greater improvement in eczema severity than placebo. Over the 6-week VGH4 period, the median SCORAD score (Table 3) decreased by 18.0 points from baseline (IQR −28.5 to −4.0), whereas during the placebo period, the median change was only −6.5 points (IQR −12.0 to 0). The within-patient difference in SCORAD improvement (VGH4 minus placebo) had a median of −10.2 points in favor of VGH4 (IQR −21.1 to +9.9). This trend approached statistical significance (paired *p* = 0.054, Wilcoxon’s signed-rank test). Notably, the magnitude of improvement with VGH4 exceeded the commonly cited minimal clinically significant difference for SCORAD (approximately 8 points) [22], suggesting a clinically meaningful benefit despite the borderline *p*-value. Furthermore, there was a greater improvement in the overall treatment effect of the SCORAD subjective score in patients treated with VGH4 compared to those treated with placebo (*p* = 0.015). No significant period or carryover effects were detected (no differences in outcomes based on treatment sequence, *p* = 0.894 for carryover) (Table 3).

Most patients experienced visible improvements during the VGH4 treatment phase, reporting reductions in erythema, lichenification, and excoriation of lesions, accompanied by decreased pruritus and better sleep quality. In contrast, during the placebo phase, some patients saw little to no improvement, and a few experienced disease flares (requiring increased use of topical steroids to control symptoms). A minority of patients improved modestly, even on placebo, reflecting the continued use of baseline therapies and natural fluctuations in AD, which contributed to the variability in outcomes. An exploratory observation was that those with the most severe baseline disease (SCORAD > 60) tended to show the most significant differential benefit from VGH4 versus placebo. However, the small sample size precludes definitive subgroup conclusions.

### 3.3. Secondary Outcome: Quality of Life

**Figure 3:**
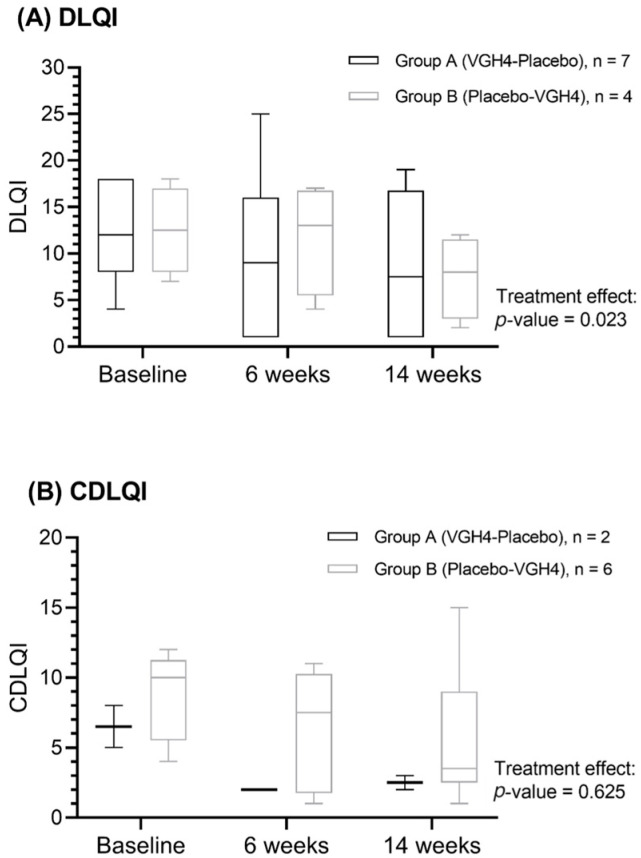
Trend of (**A**) Dermatology Life Quality Index (DLQI) and (**B**) Children’s Dermatology Life Quality Index (CDLQI) during treatment periods. The treatment effect of VGH4 compared to placebo was evaluated using the Wilcoxon signed-rank test. The DLQI score decreased after intervention in Group A; the overall treatment effect of VGH4 was statistically significant (*p* = 0.023). However, no significant differences were observed among children (*p* = 0.625).

Treatment with VGH4 resulted in significantly better quality-of-life scores than placebo. Among the 10 participants who were ≥16 years old (completing the DLQI), the median DLQI score decreased by 6 points during the VGH4 period (indicating an improvement in QoL) versus a 2-point median decrease during the placebo period (*p* = 0.023) (Figure 2). The study results indicate a greater alleviation of QoL impairment when patients underwent herbal treatment. For the eight younger participants (completing the CDLQI), QoL also improved more on VGH4 than on placebo, but the difference was not statistically significant (median improvement 4.5 vs. 3.0 points; *p* = 0.625) (Figure 3). The lack of statistical significance in the pediatric subgroup may be due to the smaller sample size and the fact that several children had low baseline CDLQI scores (indicating relatively preserved QoL despite severe dermatitis), leaving less room for measurable improvement. No carryover or period effects were observed for QoL metrics (no significant influence of treatment order on DLQI/CDLQI outcomes).

### 3.4. Immunologic Markers

Serum biomarker analyses did not reveal significant differences between the VGH4 and placebo periods. Baseline total IgE levels were markedly elevated in many participants (median ≈ 1200 IU/mL, reflecting atopic status). After 6 weeks of treatment, IgE levels showed a slight median increase of +5 IU/mL on VGH4, compared to a +20 IU/mL median change on placebo; this difference was not statistically significant (*p* = 0.193). Eosinophil counts (median ~500 cells/µL at baseline) decreased modestly over time in both groups; the median change was −0.9 × 10^9/L on VGH4 versus −0.3 × 10^9/L on placebo, with no significant difference between treatments (*p* = 0.845). Eosinophil cationic protein (ECP) levels varied among individuals, and there was no clear pattern of change attributable to VGH4 (the VGH4 vs. placebo comparison for ECP was not significant, *p* = 0.548). Overall, VGH4 did not significantly alter these systemic immunoallergic markers relative to placebo over the 6-week treatment period.

### 3.5. Safety

VGH4 was generally safe and well-tolerated in this trial. No serious adverse events (SAEs) occurred during either treatment period, and no participants discontinued the study due to adverse effects. Self-reported side effects were minimal. Two patients noted a mild, transient bitter taste immediately after taking VGH4 granules (this was only reported during the VGH4 treatment phase, likely owing to the herbs’ natural bitterness); no such complaints occurred with the placebo. There were no reports of gastrointestinal upset or any other systemic symptoms attributable to the study treatments.

Routine laboratory monitoring revealed no clinically significant abnormalities or differences between the VGH4 and placebo periods (Table 4). Liver function remained stable: for example, the mean change in alanine aminotransferase (ALT) was −1.5 U/L on VGH4 versus −1.0 U/L on placebo, and median AST changes were approximately 0 U/L in both periods (no significant differences, *p* > 0.3), with no patient developing an ALT or AST elevation above two times the upper limit of normal. Renal function was unaffected, with median changes in serum creatinine of <0.1 mg/dL in both groups, and negligible changes in BUN (*p* > 0.6 for VGH4 vs. placebo). The hematologic measurements (hemoglobin, white blood cell count with differential, and platelet count) remained within normal limits; moreover, there was no indication of bone marrow suppression or hematologic adverse effects. In this short-term trial, the safety profile of VGH4 was similar to that of the placebo.

## 4. Discussion

This randomized, crossover pilot trial suggests that adjunctive VGH4 may have potential clinical applications in moderate-to-severe atopic dermatitis; however, the primary SCORAD comparison numerically favored VGH4 without reaching the prespecified two-sided α = 0.05. VGH4 was also associated with a significant improvement in adults (*p* = 0.023), and no apparent hepatic or renal laboratory safety signals were observed during the study period [22]. However, the observed between-treatment differences in SCORAD were smaller and more variable than anticipated in the sample size calculation, resulting in limited statistical power to detect a difference at the prespecified two-sided α = 0.05.

To deliver a more refined evaluation of treatment response, we further analyzed SCORAD by distinguishing its objective and subjective components. The subjective component, which reflects patient-reported pruritus and sleep disturbance, showed a significant difference for VGH4 (*p* = 0.015). In atopic dermatitis, subjective symptoms frequently have a more direct impact on daily functioning and quality of life than objective skin lesions alone. In particular, persistent itching and sleep disturbance are among the most burdensome features reported by patients. The improvement in subjective SCORAD observed in this study, therefore, suggests that VGH4 may have influenced aspects of disease burden that are especially significant to patients. The data may also explain the improvement observed in adult DLQI. At the same time, the difference between subjective and objective components suggests that changes in patient-reported symptoms and clinician-assessed signs may not occur in parallel over a short treatment period. The results support the inclusion of symptom-focused endpoints in future confirmatory studies of VGH4.

The improvement in adult DLQI observed with VGH4 is not only statistically significant but also clinically meaningful, as it surpasses the minimum clinically important difference (MCID) of 4 points for the DLQI [23,24]. The median DLQI reduction of ~6 points during VGH4 (versus approximately 2 points on placebo) indicates a tangible improvement in QoL, exceeding the threshold for a meaningful patient benefit. Moreover, the magnitude of this QoL improvement is comparable to that achieved with established systemic therapies for moderate-to-severe AD [25]. This discrepancy may reflect the fact that, in atopic dermatitis, QoL and clinician-assessed signs are related but distinct outcome domains, and total SCORAD is influenced predominantly by lesion extent and intensity. DLQI may more readily capture short-term improvements in patient-perceived burden than the overall SCORAD, although this interpretation remains exploratory [26]. By contrast, the pediatric subgroup’s QoL changes did not reach significance (CDLQI *p* = 0.625). This lack of a significant pediatric effect may reflect the small sample size (*n* = 8) and a “floor” effect from generally low baseline CDLQI scores (several children had relatively mild QoL impairment at entry), which limited the room for measurable improvement. A lower dosage of VGH4 exposure in younger children may have contributed to the smaller observed effect. It is also possible that the CDLQI instrument is less sensitive in younger patients; notably, the MCID for the CDLQI in pediatric AD has been estimated at approximately 6 to 8 points, a higher threshold than in adults [23].

These findings suggest that VGH4 may contribute to symptom improvement in patients with AD, since it was associated with reduced AD severity and also improved overall QoL. It was used as an adjunct to standard therapy in the trial, with patients continuing their usual emollients and low-potency topical steroids, thereby suggesting that it may be compatible with a multimodal treatment regimen. The use of low-potency topical steroids was not prospectively quantified by weight, and its contribution to the observed clinical improvement cannot be precisely separated from that of VGH4.

The components of VGH4 possess established pharmacological properties that are likely to account for the observed effects. Representative candidates reported in previous studies include glycyrrhizin/glycyrrhizic acid from *Glycyrrhiza* spp., which ameliorate AD-like inflammation and reduce IgE and HMGB1/NF-kB-related signaling [27,28]; matrine from Ku-Shen (Sophora *flavescents*), which improves AD by modulating Th1/Th2 balance and inhibiting Hsp90/NF-kB signaling [29]; paeoniflorin, a major bioactive compound present in DGYZ that has also shown therapeutic effects in experimental AD [30]; and fraxinellone and dictamnine from Bai-Xian-Pi (*Dictamnus dasycarpus* Turcz.), which demonstrate anti-inflammatory and antipruritic effects in AD models [31,32].

Atopic dermatitis is characterized by type-2-skewed immune responses and eosinophilic inflammation. Systemic biomarkers such as total IgE, peripheral eosinophil counts, and eosinophil cationic protein (ECP) are commonly used to reflect underlying atopic inflammation and eosinophil activation [33,34,35]. Therefore, in this study, these markers were evaluated as exploratory indicators of systemic immunologic activity. However, circulating biomarkers may not always change in parallel with short-term improvements in skin lesions, particularly in relatively short treatment periods, and the 6-week treatment duration in this trial may have limited the ability to detect measurable changes in these systemic parameters [25,36]. The absence of significant between-treatment differences in IgE, eosinophil, and ECP should be interpreted cautiously.

This study also demonstrates a practical approach to maintaining participant blinding in herbal medicine trials. To ensure that the placebo granules closely matched the active intervention in appearance and organoleptic properties, 1% (*w*/*w*) of the active VGH4 preparation was incorporated into the placebo. Treatment allocation was known only to the dispensing pharmacist after participant enrollment, and all other investigators and participants remained blinded. However, a formal post-study blinding questionnaire was not performed, and the success of blinding was therefore not formally assessed. Blinding appeared to be preserved, as participants were unable to distinguish between the two treatments. Given that the total herbal content in the placebo was approximately 0.04 g per dose, which is less than 1/50 of the therapeutic dose, it is unlikely to exert clinically meaningful effects [37,38]. No evidence of a carryover effect was observed between treatment periods. Prior methodological work in TCM clinical research has similarly suggested that low-level “spiked” placebos may enhance the rigor of herbal trials by preserving blinding while minimizing the risk of confounding efficacy outcomes.

The sample size for this pilot study was based on a Monte Carlo simulation that assumed a within-patient treatment difference of 18 points and a standard deviation of 15 points for SCORAD. However, based on the results of this pilot study, the observed median difference in SCORAD was −10.2 points, with an estimated standard deviation of about 23 points (Table 3). This resulted in substantially lower statistical power than initially anticipated, and likely contributed to the borderline *p*-value (*p* = 0.054) observed for the primary outcome. These findings indicate that a larger study will be required to more reliably estimate treatment effects.

Several additional limitations should be acknowledged. First, this pilot study enrolled participants aged 6 to 30 years. The lower age limit was selected to ensure that participants were able to take the oral granule formulation and comply with the study procedures, whereas the upper age limit was chosen to maintain a relatively homogeneous study population and reduce clinical heterogeneity from broader adult AD populations. However, the inclusion of both pediatric and adult participants may still have introduced heterogeneity, as the clinical presentation and treatment response of AD can differ across age groups. Therefore, the generalizability of these findings to specific pediatric or adult AD populations remains limited. Second, the study did not prospectively collect Eczema Area and Severity Index (EASI), Investigator Global Assessment (IGA), Patient Global Assessment (PGA) or validated responder thresholds such as EASI-90, and these endpoints cannot be reconstructed retrospectively. Although SCORAD is a validated composite assessment scale that simultaneously captures both objective signs and patient-reported symptoms, incorporating EASI, IGA, and PGA would enable a more comprehensive and rigorous clinical evaluation and allow for a more meaningful comparison with placebo. Accordingly, treatment response could not be evaluated using the standard endpoints commonly used in contemporary AD trials, and the efficacy findings should be interpreted as preliminary. Third, all participants continued background standard care throughout the trial. Although these concomitant treatments were kept as stable as possible, some confounding of the observed treatment effects cannot be excluded, particularly because topical corticosteroid use was not prospectively quantified in a standardized manner. Fourth, the 6-week treatment duration was a pragmatic choice to capture early treatment signals, but this is shorter than the treatment duration commonly used in prior AD studies; therefore, this have underestimated the full magnitude and persistence of treatment benefits.

Even with these limitations, this pilot study lays the groundwork for future research. The observed effect size and variability support the need for a larger, adequately powered confirmatory trial. Future studies should recruit more participants, enroll age-stratified or more clinically homogeneous cohorts, prospectively quantify concomitant topical treatment, incorporate validated contemporary AD endpoints, and consider longer treatment and follow-up with systematic safety monitoring to assess the durability and possible accrual of benefit.

Prior systematic reviews suggest that TCM therapy may provide clinical benefit in AD when implemented as an evidence-based formula with monitoring for uncommon adverse events and potential herb–drug interactions [9]. However, the role of herbal preparations in evidence-based AD management remains to be fully established, particularly in pediatric populations. In addition, the pilot study was not designed or powered to detect uncommon events such as allergic sensitization or to assess longer-term allergic outcomes. Long-term studies with systematic safety monitoring are therefore warranted. In this study, VGH4 was well tolerated across the age range, with no SAEs or evidence of injury observed; larger trials are warranted to confirm these findings and further define its safety profile as a complementary therapy for AD.

## 5. Conclusions

In this randomized, double-blind, placebo-controlled crossover pilot trial, VGH4 was generally well tolerated as a short-term adjunct to standard care in moderate-to-severe AD, with no SAEs and no apparent hepatic or renal laboratory safety signals observed during the study period. Although the primary SCORAD comparison numerically favored VGH4, it did not reach the prespecified significance threshold, and efficacy cannot be established from this pilot study alone. The observed improvements in the subjective SCORAD component and in adult QoL suggest a possible effect on patient-reported symptom burden, particularly pruritus and sleep disturbance. Given the pilot nature of the study, larger and more adequately powered studies are needed to confirm efficacy and to further clarify the role of VGH4 as a complementary approach to AD management.

## Figures and Tables

**Table 1:** The composition of VGH4.

Herbal Formula	No. of Herbs	Composition	Weight per Sachet, g
Xiao-Feng-San	12	Dang-Gui (*Angelica sinensis* (Oliv.) Diels)	0.18
		Shen-Di-Huang (*Rehmannia glutinosa* (Gaertn.) Libosch. ex Fisch. et Mey.)	0.18
		Fang-Feng (*Saposhnikovia divaricata* (Turcz.) Schischk.)	0.18
		Chan-Tui (Periostracum Cicadae)	0.18
		Zhi-Mu (*Anemarrhena Rhizome*)	0.18
		Ku-Shen (*Sophora flavescens* Aiton)	0.18
		Hu-Ma-Ren (*Sesamum indicum* L.)	0.18
		Jing-Jie (*Schizonepeta tenuifolia* Briq.)	0.18
		Cang-Zhu (*Atractylodes chinensis* (DC.) Koidz.)	0.18
		Niu-Bang-Zi (*Arctium lappa* L.)	0.18
		Shih-Gao (Gypsum Fibrosum)	0.18
		Gan-Cao (*Glycyrrhiza uralensis* Fisch.)	0.09
		Mu-Tong (*Akebia quinata* (Thunb.) Decne.)	0.09
Dang-Gui-Yin-Zi	11	Dang-Gui (*Angelica sinensis* (Oliv.) Diels)	0.12
		Bai-Shao (*Paeonia lactiflora* Pall.)	0.12
		Chuan-Qiong (*Ligusticum chuanxiong* Hort.)	0.12
		Shen-Di-Huang (*Rehmannia glutinosa* (Gaertn.) Libosch. ex Fisch. et Mey.)	0.12
		Bai-Ji-Li *(Tribulus cuneatus* Sieb. et Zucc.)	0.12
		Fang-Feng (*Saposhnikovia divaricata* (Turcz.) Schischk.)	0.12
		Jing-Jie (*Schizonepeta tenuifolia* Briq.)	0.12
		He-Shou-Wu (*Polygonum multiflorum* Thunb.)	0.06
		Huang-Qi (*Astragalus membranaceus* (Fisch.) Bunge)	0.06
		Gan-Cao (*Glycyrrhiza uralensis* Fisch.)	0.06
		Sheng-Jiang (*Zingiber officinale* Roscoe)	0.18
Single herbs	2	Bai-Xian-Pi (*Dictamnus dasycarpus Turcz.*)	0.4
		Di-Fu-Zi (*Kochia scoparia* (L.) Schrad.)	0.5

VGH4 is a combination of Xiao-Feng-San, Dang-Gui-Yin-Zi, Bai-Xian-Pi, and Di-Fu-Zi, with the ratio of 2 gm, 1.2 gm, 0.4 gm, and 0.5 gm, respectively.

**Table 2:** Baseline characteristics of study participants (*n* = 19).

		Group A (VGH4–Placebo) (*n* = 9)	Group B (Placebo–VGH4) (*n* = 10)	*p*-Value ^a^
		*n* (%)	*n* (%)	
Gender				0.650
	Female	6 (66.67%)	5 (50.00%)	
	Male	3 (33.33%)	5 (50.00%)	
Age (year), median (Q1–Q3)		17.75 (16.10–26.40)	15.53 (11.82–24.57)	0.488
Accompanying allergic disease (%)				
	Asthma	4 (44.44%)	1 (10.00%)	0.141
	Allergic rhinitis	7 (77.78%)	7 (70.00%)	1.000
	Urticaria	3 (33.33%)	5 (50.00%)	0.650
	Food allergy	6 (66.67%)	7 (70.00%)	1.000
Family medical history (%)				
	Atopic dermatitis	2 (22.22%)	2 (20.00%)	1.000
	Asthma	2 (22.22%)	5 (50.00%)	0.350
	Allergic rhinitis	8 (88.89%)	8 (80.00%)	1.000
	Urticaria	1 (11.11%)	4 (40.00%)	0.303
	Food allergy	3 (33.33%)	4 (40.00%)	1.000
Treatment history (%)				
	Antihistamine	7 (77.78%)	8 (80.00%)	1.000
	Topical corticosteroid	8 (88.89%)	8 (80.00%)	1.000
	Oral steroid	6 (66.67%)	4 (40.00%)	0.370
	Intralesional steroid	2 (22.22%)	0 (0.00%)	0.211
	Phototherapy	3 (33.33%)	1 (10.00%)	0.303
	Acupuncture	4 (44.44%)	5 (50.00%)	1.000
SCORAD, median (Q1–Q3)				
	Total score	54.10 (52.40–65.30)	50.25 (43.45–60.05)	0.377
	Subjective score	12.00 (8.00–14.00)	13.00 (12.00–15.00)	0.505
DLQI/CDLQI, median (Q1–Q3)				
	DLQI	12.00 (8.00–18.00)	12.50 (9.00–16.00)	0.924
	CDLQI	6.50 (5.00–8.00)	10.00 (6.00–11.00)	0.402

^a^ Fisher’s exact test was performed to examine differences in percentages for categorical variables. Wilcoxon’s rank-sum test was performed for continuous variables. Abbreviations: SCORAD, SCOring Atopic Dermatitis. DLQI, Dermatology Life Quality Index. CDLQI, Children’s Dermatology Life Quality Index.

**Table 3:** Treatment effects of VGH4 on SCOring Atopic Dermatitis Index (SCORAD).

	Treatment Period	
	Period 1	Period 2
Total SCORAD		
Group A (VGH4–Placebo)		
Median (Q1–Q3)	−31.13 (−38.25 to −21.08)	−19.05 (−43.28 to −5.55)
Group B (Placebo–VGH4)		
Median (Q1–Q3)	−7.08 (−14.45 to 1.35)	−12.8 (−33.35 to −1.6)
Treatment effect (VGH4 vs. Placebo)		
Median (Q1–Q3)	−10.2 (−21.1 to 9.9)	
*p*-value ^a^	0.054	
SCORAD subscore		
Objective treatment effect (VGH4 vs. Placebo)		
Median (Q1–Q3)	−5.1 (−18.1 to 7.9)	
*p*-value ^a^	0.196	
Subjective treatment effect (VGH4 vs. Placebo)		
Median (Q1–Q3)	−3.0 (−6.0 to 1.0)	
*p*-value ^a^	0.015 *	
Carryover effect		
*p*-value ^b^	0.894	

^a^ Wilcoxon’s signed-rank test was performed to examine the change in SCORAD score after intervention. Placebo was treated as the referent group. ^b^ Wilcoxon’s rank-sum test was performed to examine difference in median between Group A and Group B. Abbreviations: SCORAD, SCOring Atopic Dermatitis Index. * *p* < 0.05.

**Table 4:** Comparisons of safety and immunobiological markers between treatment groups.

	Allocation Sequence AB (VGH4–Placebo)		Allocation Sequence BA (Placebo–VGH4)		*p*-Value ^a^
	Group-Level Median (Q1–Q3)		Group-Level Median (Q1–Q3)		
	Period 1	Period 2	Period 1	Period 2	
	VGH4	Placebo	Placebo	VGH4	
Safety					
BUN	−1 (−3 to 0)	−2.5 (−3.5 to 1)	−1.5 (−2 to 1)	0.5 (−1 to 1)	0.958
Crea	0.01 (−0.03 to 0.05)	−0.02 (−0.05 to 0.06)	0.05 (−0.01 to 0.08)	0.07 (−0.02 to 0.1)	0.671
ALT	−3 (−10 to 1)	−3 (−7.5 to 1.5)	−3.5 (−7 to 0)	−4.5 (−7 to −1)	0.310
AST	−2 (−7 to −1)	1.5 (−5.5 to 5)	−4 (−6 to −2)	−2 (−5 to 1)	0.934
Immunobiology markers					
IgE	−61 (−99 to 404)	−108 (−382.35 to 301.5)	18.5 (−59 to 630.5)	150.5 (−61 to 524.5)	0.193
ECP	−11.2 (−17.2 to 6.34)	−9.05 (−20.1 to 7.05)	−1.67 (−11.7 to 1.1)	0.12 (−14.8 to 8.8)	0.548
EOS	−0.9 (−1.5 to −0.4)	−0.3 (−2.2 to 1.1)	−0.25 (−2.1 to 3.5)	−0.3 (−3.4 to 3.2)	0.845

^a^ *p*-values were calculated by Wilcoxon’s signed-rank test to examine the changes in values after intervention. Abbreviations: BUN, blood urea nitrogen; Crea, creatinine; ALT, alanine aminotransferase; AST, aspartate aminotransferase; ECP, eosinophil cationic protein; EOS, eosinophil counts.

## Data Availability

Data can be obtained from the authors upon reasonable request.

## Supplementary Materials

The following supporting information can be downloaded at https://www.mdpi.com/article/10.3390/life16040680/s1, File S1: Quality Control and Certificates of Analysis (CoA) for VGH4 composition. The original reports are issued in Chinese; Table S1 provides an English index and summary. Table S1: Index of Certificates of Analysis (CoA) and key quality control tests for VGH4 composition. File S2: Stability testing of finished products (VGH4 and placebo) The original reports are issued in Chinese; Table S2 provides an English index and summary. Table S2: Index of accelerated stability testing reports and key test items for finished products.

## Abbreviations

The following abbreviations are used in this manuscript:

AD	Atopic dermatitis
TCM	Traditional Chinese medicine
QoL	Quality of life
NHIRD	National Health Insurance Research Database
XFS	Xiao-Feng-San
DGYZ	Dang-Gui-Yin-Zi
QC	Quality Control
SCORAD	SCOring Atopic Dermatitis Index
DLQI	Dermatology Life Quality Index
CDLQI	Children Dermatology Life Quality Index
SAEs	Serious adverse events
ECP	Eosinophil cationic protein
BUN	Blood urea nitrogen
ALT	Alanine aminotransferase
AST	Aspartate aminotransferase
MCID	Minimum clinically important difference
EASI	Eczema Area and Severity Index
IGA	Investigator Global Assessment
PGA	Patient Global Assessment
