# Supplementary material for: Adjunctive Use of VGH4 for Moderate-to-Severe Atopic Dermatitis: A Randomized, Double-Blind, Placebo-Controlled Crossover Pilot Trial
Source: Life (Basel). 2026 Apr 16;16(4):680. doi: 10.3390/life16040680 (PMC13117100; doi:10.3390/life16040680)
Supplement: Supplementary file 1 [file life-16-00680-s001.zip › Supplementary File S1 & File S2.pdf]

Supplementary File S1. Quality Control and Certificates of Analysis (CoA) for VGH4 composition. The original reports are issued in Chinese; Table S1 provides an English index and summary.

Table S1. Index of Certificates of Analysis (CoA) and key quality control tests for VGH4 composition.

Section	Formula Name (English)	Batch/Lot No.	Key tests included	Summary of results
S1.1	Xiao-Feng-San concentrated powder	31029805	TLC identification; marker-based assay; microbiological limits; heavy metals (As/Pb/Cd/Hg); other physicochemical tests	Within specification / Pass; TLC identification reported as compliant
S1.2	Dang-Gui-Yin-Zi concentrated granules	32127804	TLC identification; microbiological limits; heavy metals (As/Pb/Cd/Hg); other physicochemical tests	Within specification / Pass; TLC identification reported as compliant
S1.3	Bai-Xian-Pi concentrated granules	420529803	TLC identification; microbiological limits; heavy metals (As/Pb/Cd/Hg); other physicochemical tests	Within specification / Pass; TLC identification reported as compliant
S1.4	Di-Fu-Zi concentrated granules	420617802	TLC identification; microbiological limits; heavy metals (As/Pb/Cd/Hg); other physicochemical tests	Within specification / Pass; TLC identification reported as compliant

Section S1.1. Xiao-Feng-San (消風散) concentrated powder (Batch No. 31029805) Certificate of Analysis (CoA)

科達製藥股份有限公司
G.M.P. KO DA PHARMACEUTICAL CO., LTD.

產品檢驗成績書
Certificate of Analysis

地址：桃園市平鎮區工業三路20-1號
Address: No. 20-1, Gongye 3rd Rd., Pingzhen Dist., Taoyuan, Taiwan
電話：03-4696105
Tel: +886-3-4696105

樣品名稱："科達"消風散濃縮散
Product Name: "Ko Da" Xiao-Feng-San concentrated powder
樣品批號：31029805
Batch No.: 31029805
產品有效日期：2022.10.15 Expiry Date: October 15, 2022

報告編號：F31029805
Report No.: F31029805
報告日期：2019.10.29
Report Date: October 29, 2019
頁　數：1/2 Page: 1/2

檢驗項目<br>DETERMINATION	判定基準<br>SPECIFICATIONS	檢驗結果<br>RESULT	定量極限<br>LOQ	方法依據<br>TEST METHOD
一般檢查 General Information	Light brown fine granules; slight odor; slightly bitter taste			
性狀 Appearance	淺黃褐色粉末，氣香，味苦微甜	Conform	-	KODA
乾燥減重 Loss on drying	≦8.0%	4.37%	-	THP
灰分 Total ash	≦10.0%	6.43%	-	THP
酸不溶性灰分 Acid-insoluble ash	≦6.0%	0.95%	-	THP
稀醇抽提物 Water extract	≧43.0%	54.78%	-	THP
水抽提物 Diluted ethanol extract	≧47.0%	57.19%	-	THP
T.L.C.鑑別 TLC identification				
甘草 Gan-Cao	Positive	Positive	-	KODA
生地黃 Shen-Di-Huang	Positive	Positive	-	KODA
防風 Fang-Feng	Positive	Positive	-	KODA
知母 Zhi-Mu	Positive	Positive	-	KODA
苦參 Ku-Shen	Positive	Positive	-	KODA
當歸 Dang-Gui	Positive	Positive	-	KODA
蒼朮 Cang-Zhu	Positive	Positive	-	KODA
含量測定 Assay				
牛蒡子苷(Arctiin)	13.5~40.5mg/day	33.02mg/day	-	KODA
甘草酸(Glycyrrhizin)	12.5~37.5mg/day	35.00mg/day	-	KODA
微生物 Microbiological Tests				
總生菌數(TAMC)	≦1.0×10^5CFU/g	Pass	-	USP
大腸桿菌(*E. coli*)	Negative	Negative	-	USP
沙門氏菌(*Salmonella*)	Negative	Negative	-	USP
重金屬測定 Heavy Metals				
總重金屬 Total heavy metals	≦30ppm	Pass	10ppm	THP

<續下頁>
Continue

科達製藥股份有限公司
G.M.P. KO DA PHARMACEUTICAL CO., LTD.

產品檢驗成績書
Certificate of Analysis

地址：桃園市平鎮區工業三路20-1號
Address: No. 20-1, Gongye 3rd Rd., Pingzhen Dist., Taoyuan, Taiwan
電話：03-4696105
Tel: +886-3-4696105

檢驗項目<br>DETERMINATION	判定基準<br>SPECIFICATIONS	檢驗結果<br>RESULT	定量極限<br>LOQ	方法依據<br>TEST METHOD
鉛 Lead (Pb)	≦10.0ppm	0.477ppm	0.1ppm	THP
砷 Arsenic (As)	≦3.0ppm	N.D.	0.25ppm	THP
鎘 Cadmium (Cd)	≦0.5ppm	0.067ppm	0.025ppm	THP
汞 Mercury (Hg)	≦0.5ppm	0.029ppm	0.025ppm	THP

品保主管：Jinming Chang
QA Supervisor:

科達製藥檢驗中心 檢驗報告專用章 TEL：+886-3-4696105 FAX：+886-3-4690546 桃園市平鎮區工業三路20-1號

N.D.：檢驗結果小於定量極限
N.D.: Not detected (below the limit of quantification, LOQ)
THP：台灣中藥典
THP: Taiwan Herbal Pharmacopoeia
KODA：科達檢驗方法
KODA: Ko Da in-house testing method
USP：美國藥典
USP: United States Pharmacopeia

Section S1.2. Dang-Gui-Yin-Zi (當歸飲子) concentrated granules (Batch No. 32127804) Certificate of Analysis (CoA)

科達製藥股份有限公司
G.M.P. KO DA PHARMACEUTICAL CO., LTD.

產品檢驗成績書
Certificate of Analysis

地址：桃園市平鎮區工業三路20-1號
Address: No. 20-1, Gongye 3rd Rd., Pingzhen Dist., Taoyuan, Taiwan
電話：03-4696105
Tel: +886-3-4696105

樣品名稱："科達"當歸飲子濃縮細粒
Product Name: "Ko Da" Dang-Gui-Yin-Zi Concentrated Granules
樣品批號：32127804
Batch No.: 32127804
產品有效日期：2021.11.10 Expiry Date: November 10, 2021

報告編號：F32127804
Report No.: F32127804
報告日期：2019.11.22
Report Date: November 22, 2019
頁　數：1/1 Page: 1/1

檢驗項目 DETERMINATION	判定基準 SPECIFICATIONS	檢驗結果 RESULT	定量極限 LOQ	方法依據 TEST METHOD
一般檢查 General Information				
性狀 Appearance	Brown fine granules with a fragrant odor; sweet with a slightly bitter taste. 棕色細粒，氣香，味甘甜微苦	Conform	-	KODA
乾燥減重 Loss on drying	≦8.0%	3.67%	-	THP
灰分 Total ash	≦9.0%	4.13%	-	THP
酸不溶性灰分 Acid-insoluble ash	≦3.0%	0.37%	-	THP
稀醇抽提物 Water extract	≧27.0%	50.41%	-	THP
水抽提物 Diluted ethanol extract	≧32.0%	54.05%	-	THP
T.L.C.鑑別 TLC identification				
川芎、當歸 Chuan-Qiong, Dang-Gui	Positive	Positive	-	KODA
甘草 Gan-Cao	Positive	Positive	-	KODA
生薑 Sheng-Jiang	Positive	Positive	-	KODA
生地黃 Shen-Di-Huang	Positive	Positive	-	KODA
白芍 Bai-Shao	Positive	Positive	-	KODA
微生物 Microbiological Tests				
總生菌數(TAMC)	≦1.0×10^5CFU/g	Pass	-	USP
大腸桿菌(*E. coli*)	Negative	Negative	-	USP
沙門氏菌(*Salmonella*)	Negative	Negative	-	USP
重金屬測定 Heavy Metals				
總重金屬 Total heavy metals	≦30ppm	Pass	10ppm	THP
鉛 Lead (Pb)	≦10.0ppm	0.219ppm	0.1ppm	THP
砷 Arsenic (As)	≦3.0ppm	N.D.	0.25ppm	THP
鎘 Cadmium (Cd)	≦0.5ppm	0.033ppm	0.025ppm	THP
汞 Mercury (Hg)	≦0.5ppm	N.D.	0.025ppm	THP

品保主管：Jinming Chang
QA Supervisor:

科達製藥檢驗中心
檢驗報告專用章
TEL：+886-3-4696105
FAX：+886-3-4690546
桃園市平鎮區工業三路20-1號

N.D.：檢驗結果小於定量極限
N.D.: Not detected (below the limit of quantification, LOQ)
THP：台灣中藥典
THP: Taiwan Herbal Pharmacopoeia
USP：美國藥典
USP: United States Pharmacopeia
KODA：科達檢驗方法
KODA: Ko Da in-house testing method

Section S1.3. Bai-Xian-Pi (白鮮皮) concentrated granules (Batch No. 420529803) Certificate of Analysis (CoA)

科達製藥股份有限公司
G.M.P. **KO DA PHARMACEUTICAL CO., LTD.**

產品檢驗成績書
Certificate of Analysis

地址：桃園市平鎮區工業三路20-1號
Address: No. 20-1, Gongye 3rd Rd., Pingzhen Dist., Taoyuan, Taiwan
電話：03-4696105
Tel: +886-3-4696105

樣品名稱："科達"白鮮皮濃縮細粒
Product Name: "Ko Da" Bai-Xian-Pi Concentrated Granules
樣品批號：420529803
Batch No.: 420529803
產品有效日期：2022.10.03 Expiry Date: October 03, 2022

報告編號：F420529803
Report No.: F420529803
報告日期：2019.10.18
Report Date: October 18, 2019
頁　數：1/1 Page: 1/1

檢驗項目<br>DETERMINATION	判定基準<br>SPECIFICATIONS	檢驗結果<br>RESULT	定量極限<br>LOQ	方法依據<br>TEST METHOD
一般檢查 General Information				
性狀 Appearance	Light brown fine granules with a slight characteristic odor; slightly bitter and cooling in taste.<br>淺褐色細粒，氣膻，味微苦、清涼	Conform	-	KODA
乾燥減重 Loss on drying	≦8.0%	3.59%	-	THP
灰分 Total ash	≦5.0%	1.17%	-	THP
酸不溶性灰分 Acid-insoluble ash	≦2.0%	0.04%	-	THP
稀醇抽提物 Water extract	≧38.0%	47.03%	-	THP
水抽提物 Diluted ethanol extract	≧44.0%	60.31%	-	THP
T.L.C.鑑別 TLC identification				
白鮮皮 Bai-Xian-Pi	Positive	Positive	-	KODA
微生物 Microbiological Tests				
總生菌數(TAMC)	≦1.0×10^5CFU/g	Pass	-	USP
大腸桿菌(*E. coli*)	Negative	Negative	-	USP
沙門氏菌(*Salmonella*)	Negative	Negative	-	USP
重金屬測定 Heavy Metals				
總重金屬 Total heavy metals	≦30ppm	Pass	10ppm	THP
鉛 Lead (Pb)	≦10.0ppm	N.D.	0.1ppm	THP
砷 Arsenic (As)	≦3.0ppm	N.D.	0.25ppm	THP
鎘 Cadmium (Cd)	≦0.5ppm	N.D.	0.025ppm	THP
汞 Mercury (Hg)	≦0.5ppm	N.D.	0.025ppm	THP

品保主管：Jinming Chang
QA Supervisor:

N.D.：檢驗結果小於定量極限
N.D.: Not detected (below the limit of quantification, LOQ)
THP ：台灣中藥典
THP: Taiwan Herbal Pharmacopoeia
USP ：美國藥典
USP: United States Pharmacopeia

KODA：科達檢驗方法
KODA: Ko Da in-house testing method

科達製藥檢驗中心
檢驗報告
專用章
TEL：+886-3-4696105
FAX：+886-3-4690546
桃園市平鎮區工業三路20-1號

Section S1.4. Di-Fu-Zi (地膚子) concentrated granules (Batch No. 420529803) Certificate of Analysis (CoA)

科達製藥股份有限公司

G.M.P. KO DA PHARMACEUTICAL CO., LTD.

產品檢驗成績書
Certificate of Analysis

地址：桃園市平鎮區工業三路20-1號
Address: No. 20-1, Gongye 3rd Rd., Pingzhen Dist., Taoyuan, Taiwan
電話：03-4696105
Tel: +886-3-4696105

樣品名稱："科達"地膚子濃縮細粒
Product Name: "Ko Da" Di-Fu-Zi Concentrated Granules
樣品批號：420617802
Batch No.: 420617802
產品有效日期：2022.10.22 Expiry Date: October 22, 2022

報告編號：F420617802
Report No.: F420617802
報告日期：2019.11.05
Report Date: November 5, 2019
頁　數：1/1 Page: 1/1

檢驗項目 DETERMINATION	判定基準 SPECIFICATIONS	檢驗結果 RESULT	定量極限 LOQ	方法依據 TEST METHOD
一般檢查 General Information				
性狀 Appearance<br>Appearance	Light brown fine granules; slight odor; slightly bitter taste<br>淺褐色細粒，氣微，味微苦	Conform	-	KODA
乾燥減重 Loss on drying	≦8.0%	2.60%	-	THP
灰分 Total ash	≦10.0%	3.32%	-	THP
酸不溶性灰分 Acid-insoluble ash	≦4.0%	0.39%	-	THP
稀醇抽提物 Water extract	≧18.0%	31.20%	-	THP
水抽提物 Diluted ethanol extract	≧36.0%	47.48%	-	THP
T.L.C.鑑別 TLC identification				
地膚子 Di-Fu-Zi	Positive	Positive	-	KODA
微生物 Microbiological Tests				
總生菌數(TAMC)	≦1.0×10^{5}CFU/g	Pass	-	USP
大腸桿菌(*E. coli*)	Negative	Negative	-	USP
沙門氏菌(*Salmonella*)	Negative	Negative	-	USP
重金屬測定 Heavy Metals				
總重金屬 Total heavy metals	≦30ppm	Pass	10ppm	THP
鉛 Lead (Pb)	≦10.0ppm	N.D.	0.1ppm	THP
砷 Arsenic (As)	≦3.0ppm	N.D.	0.25ppm	THP
鎘 Cadmium (Cd)	≦0.5ppm	N.D.	0.025ppm	THP
汞 Mercury (Hg)	≦0.5ppm	N.D.	0.025ppm	THP

品保主管：
QA Supervisor: Jinming Chang

N.D.：檢驗結果小於定量極限
N.D.: Not detected (below the limit of quantification, LOQ)
THP ：台灣中藥典
THP: Taiwan Herbal Pharmacopoeia
USP ：美國藥典
USP: United States Pharmacopeia

KODA：科達檢驗方法
KODA: Ko Da in-house testing method

科達製藥檢驗中心
檢驗報告專用章
TEL：+886-3-4696105
FAX：+886-3-4690546
桃園市平鎮區工業三路20-1號

Supplementary File S2. Stability testing of finished products (VGH4 and placebo) The original reports are issued in Chinese; Table S2 provides an English index and summary.

Table S2. Index of accelerated stability testing reports and key test items for finished products

**Section**	**Finished product**	**Batch/Lot No.**	**Stability design / timepoints**	**Key tests included**	**Summary of results**
S2.1	VGH4	A10714901	Accelerated stability at 0/3/6 months	Appearance/organoleptic; physicochemical tests (loss on drying, extract content); microbiological limits (total aerobic microbial count); TLC identification for formula components	Within specification / Pass across reported timepoints; TLC identification reported as positive/compliant at all timepoints; microbiological limits within specification
S2.2	Placebo	A10715901	Accelerated stability at 0/3/6 months	Appearance/organoleptic; physicochemical tests (loss on drying, extract content); microbiological limits (total aerobic microbial count); identification tests	Within specification / Pass across reported timepoints; microbiological limits within specification

Section S2.1. VGH4 (Batch No. A10714901) accelerated stability test report

科達製藥股份有限公司 KO DA PHARMACEUTICAL CO., LTD.

成品安定性試驗表（一）加速試驗 Stability Study Report (I): Accelerated Testing

品名 Product Name	"北榮"異位性皮膚炎中藥 "VGHTPE" Traditional Chinese Medicine (TCM) for atopic dermatitis			批量 Batch Size	9.84 kg
包裝型態 Packaging	4.1g 銀色方形鋁箔包 4.1 g silver square aluminum foil sachets	試驗間隔 Quantity	0M、3M、6M	儲存條件 Storage Conditions	溫度 Temperature：40±2 ℃<br>濕度 Humidity：75±5 %RH
數量 Quantity	12×2 包 12 × 2 sachets	檢驗方法 Test Method	F A10714	劑型 Dosage Form	濃縮顆粒劑 Concentrated granules
製造批號 Product	A10714901	製造日期 Manufacturing Date	109.2.13 February 13, 2020	入庫日期 Storage Date	109.2.25 February 25, 2020

試驗間隔 Testing Intervals<br>試驗項目 Test Item	判定基準 Specification	第 0 月 0 Month (0M)<br>February 14, 2020<br>109 年 2 月 14 日	第 3 月 3 Month (3M)<br>May 25, 2020<br>109 年 5 月 25 日	第 6 月 6 Month (6M)<br>August 25, 2020<br>109 年 8 月 25 日
性狀 Appearance	非生霉腐敗之乾燥細粒 Dry fine granules without mold growth or deterioration.	符合規定 Conform	符合規定 Conform	符合規定 Conform
顏色 Color	褐色細粒 Brown fine granules	符合規定 Conform	符合規定 Conform	符合規定 Conform
乾燥減重 Loss on drying	8.00 %以下 ≤ 8.00%	2.47 %	2.60 %	2.79 %
稀醇抽提物 Diluted ethanol extract	38.00 %以上 ≥ 38.00%	48.14 %	48.22 %	48.34 %
水抽提物 Water extract	49.00 %以上 ≥ 49.00%	58.51 %	59.62 %	59.72 %
T.L.C.鑑別 TLC identification	消風散濃縮散：Rf 值約 0.36 附近有一黃綠色點<br>Xiao-Feng-San concentrated powder: a yellowish-green spot is observed at an Rf value of approximately 0.36.	Positive	Positive	Positive
T.L.C.鑑別 TLC identification	當歸飲子濃縮細粒：Rf 值約 0.68 附近有一紫色點<br>Dang-Gui-Yin-Zi concentrated granules: a purple spot is observed at an Rf value of approximately 0.68.	Positive	Positive	Positive
總生菌數 Total aerobic microbial count	10^5 CFU/g 以下 ≤ 1.0 × 10^5 CFU/g	< 1.0×10^2 CFU/g	< 1.0×10^2 CFU/g	1.0×10^2 CFU/g
判定 Result		合格 Pass	合格 Pass	合格 Pass

Section S2.2. Placebo (Batch No. A10714901) accelerated stability test report

科達製藥股份有限公司 KO DA PHARMACEUTICAL CO., LTD.

成品安定性試驗表（一）加速試驗 Stability Study Report (I): Accelerated Testing

品名 Product Name	"北榮"異位性皮膚炎安慰劑 "VGHTPE" Atopic Dermatitis Placebo			批量 Batch Size	50.02 kg
包裝型態 Packaging	4.1g 銀色方形鋁箔包 4.1 g silver square aluminum foil sachets	試驗間隔 Quantity	0M、3M、6M	儲存條件 Storage Conditions	溫度 Temperature：40±2 ℃ 濕度 Humidity：75±5 %RH
數量 Quantity	12×2 包 12 × 2 sachets	檢驗方法 Test Method	F A10715	劑型 Dosage Form	濃縮顆粒劑 Concentrated granules
製造批號 Product	A10715901	製造日期 Manufacturing Date	109.3.26 March 26, 2020	入庫日期 Storage Date	109.4.14 April 14, 2020

試驗間隔 Testing Intervals 試驗項目 Test Item	判定基準 Specification	第 0 月 0 Month (0M) March 31, 2020 109 年 3 月 31 日	第 3 月 3 Month (3M) July 14, 2020 109 年 7 月 14 日	第 6 月 6 Month (6M) October 14, 2020 109 年 10 月 14 日
性 狀 Appearance	非生霉腐敗之乾燥細粒 Dry fine granules without mold growth or deterioration.	符合規定 Conform	符合規定 Conform	符合規定 Conform
顏 色 Color	褐色細粒 Brown fine granules	符合規定 Conform	符合規定 Conform	符合規定 Conform
乾燥減重 Loss on drying	8.00 %以下 ≤ 8.00%	2.33 %	3.00 %	3.12 %
稀醇抽提物 Diluted ethanol extract	23.00 %以上 ≥ 23.00%	32.67 %	33.12 %	33.93 %
水抽提物 Water extract	28.00 %以上 ≥ 49.00%	38.14 %	37.98 %	38.74 %
總生菌數 Total aerobic microbial count	10^5 CFU/g 以下 ≤ 1.0 × 10^5 CFU/g	1.0×10^2 CFU/g	1.5×10^2 CFU/g	2.0×10^2 CFU/g
判定 Result		合 格 Pass	合 格 Pass	合 格 Pass
